# Effect of nitrogen availability on the poly-3-d-hydroxybutyrate accumulation by engineered *Saccharomyces cerevisiae*

**DOI:** 10.1186/s13568-017-0335-z

**Published:** 2017-02-07

**Authors:** Diogo J. Portugal-Nunes, Sudhanshu S. Pawar, Gunnar Lidén, Marie F. Gorwa-Grauslund

**Affiliations:** 1 Applied Microbiology, Department of Chemistry, Lund University, PO Box 124, 221 00 Lund, Sweden; 2 Department of Chemical Engineering, Lund University, PO Box 124, 221 00 Lund, Sweden

**Keywords:** Poly-3-d-hydroxybutyrate, PHB, Bioplastic, *Saccharomyces cerevisiae*, Nitrogen limitation, Glycogen

## Abstract

Poly-3-d-hydroxybutyrate (or PHB) is a polyester which can be used in the production of biodegradable plastics from renewable resources. It is naturally produced by several bacteria as a response to nutrient starvation in the excess of a carbon source. The yeast *Saccharomyces cerevisiae* could be an alternative production host as it offers good inhibitor tolerance towards weak acids and phenolic compounds and does not depolymerize the produced PHB. As nitrogen limitation is known to boost the accumulation of PHB in bacteria, the present study aimed at investigating the effect of nitrogen availability on PHB accumulation in two recombinant *S. cerevisiae* strains harboring different xylose consuming and PHB producing pathways: TMB4443 expressing an NADPH-dependent acetoacetyl-CoA reductase and a wild-type *S. stipitis* XR with preferential use of NADPH and TMB4425 which expresses an NADH-dependent acetoacetyl-CoA reductase and a mutated XR with a balanced affinity for NADPH/NADH. TMB4443 accumulated most PHB under aerobic conditions and with glucose as sole carbon source, whereas the highest PHB concentrations were obtained with TMB4425 under anaerobic conditions and xylose as carbon source. In both cases, the highest PHB contents were obtained with high availability of nitrogen. The major impact of nitrogen availability was observed in TMB4425, where a 2.7-fold increase in PHB content was obtained. In contrast to what was observed in natural PHB-producing bacteria, nitrogen deficiency did not improve PHB accumulation in *S. cerevisiae*. Instead the excess available carbon from xylose was shunted into glycogen, indicating a significant gluconeogenic activity on xylose.

## Introduction

Poly-3-d-hydroxybutyrate (PHB) was the first discovered microbial produced alkanoate (Khanna and Srivastava 2005). PHB is one of the best candidates for production of fully biodegradable plastics from renewable resources as it possesses similar characteristics as the most common plastics used in industry—polyethylene and polypropylene (Khanna and Srivastava 2005; Wang and Lee 1997). PHBs are also thermoplastic and biocompatible, which opens the possibility for medical applications (Verlinden et al. 2007; Yoneyama et al. 2015).

These compounds are naturally accumulated as intracellular carbon granules and energy storage material in several bacteria, the so-called ‘natural producers’, such as *Cupriavidus necator*, *Bacillus megaterium* and several species belonging to the genus *Pseudomonas* (Trotsenko and Belova 2000; Verlinden et al. 2007). PHB production from the acetyl coenzyme A (acetyl-CoA) intermediate occurs in a three step process consisting of (a) an acetyl-CoA acetyltransferase, which catalyzes the combination of two acetyl-CoA to acetoacetyl-CoA (Peoples and Sinskey 1989b), (b) an acetoacetyl-CoA reductase, which reduces acetoacetyl-CoA to 3-d-hydroxybutyryl-CoA (Peoples and Sinskey 1989b) and (c) a PHB synthase, catalyzing the final polymerization step (Peoples and Sinskey 1989a). The accumulation of PHB is generally optimal under excess of carbon and limiting levels of nitrogen, phosphorous and/or oxygen (Verlinden et al. 2007).

Although the natural producers are reported to accumulate more than 60% PHB per cell biomass (Budde et al. 2011; Schlegel et al. 1961), growth is slow and the PHB recovery might be challenging (Suriyamongkol et al. 2007). Moreover, the presence of endogenous depolymerases that can utilize the accumulated PHB as carbon/energy, reduces the overall production (Jendrossek and Handrick 2002). The price of the raw materials—so far sugars of agricultural origin—and their transportation can be two major contributors for the final cost. So, despite the numerous advantages compared to the traditional oil-based plastics, commercial bio-based PHB production has not been reported yet. Sugars derived from lignocellulosic biomass or other sugar-rich waste streams are alternative carbon sources, which have environmental advantages in terms of a reduced net release of carbon dioxide in comparison to crop-derived sugars (Dornburg et al. 2008). Hydrolysates from lignocellulosic biomass are composed of a mixture of several monomeric sugars, which can be used as carbon sources by microorganisms (Olsson and Hahn-Hägerdal 1996). For instance, spent sulfite liquor (SSL) is a sugar-rich stream generated by the sulfite based pulp-mills (Inskeep et al. 1951) that has been extensively studied over the past decades as a potential underutilized source of sugars. The relative proportion of monosaccharides in the SSL will strongly depend on the feedstock, where softwood—such as spruce and pine—will give high proportions of hexoses such as mannose and glucose, whereas SSL from hardwoods—such as Eucalyptus—will contain predominantly xylose (Lawford and Rousseau 1993) [see also recent review from (Pereira et al. 2013)]. However, a significant challenge with lignocellulosic feedstock is the presence, after hydrolysis, of weak acids and phenolic compounds that act as microbial inhibitors (Olsson and Hahn-Hägerdal 1996; Palmqvist and Hahn-Hägerdal 2000; Taherzadeh et al. 1997).

As an alternative to natural hosts, PHB pathways from natural PHB producers have been introduced in organisms that grow fast, cannot re-use accumulated PHB as a carbon source or are able to resist inhibitors. The bacterium *Escherichia coli* (Fidler and Dennis 1992; Kalousek and Lubitz 1995), the yeast *Saccharomyces cerevisiae* (Breuer et al. 2002; Kocharin et al. 2012; Leaf et al. 1996; Sandström et al. 2015), insect cells of armyworm and plants like tobacco (Poirier et al. 1992; Suriyamongkol et al. 2007) are some of the prominent examples. When using lignocellulose-derived sugars as raw materials, *S. cerevisiae* is probably one of the most suitable candidate host due to its fermentation capacity, robustness and extensive characterization (Olsson and Hahn-Hägerdal 1993; Ostergaard et al. 2000). PHB production from glucose was first attempted in *S. cerevisiae* through the expression of the PHB synthase gene (Leaf et al. 1996) and further boosted by expressing the acetyl-CoA acetyltransferase and acetoacetyl-CoA reductase genes from *C. necator* (Breuer et al. 2002; Carlson and Srienc 2006). PHB accumulation was further improved by increasing the availability of the precursor acetyl-CoA and the pool of the co-factor nicotinamide adenine dinucleotide phosphate (NADPH) (Kocharin et al. 2012, 2013). More recently, PHB formation from xylose as the sole carbon source was obtained under aerobic conditions in an engineered *S. cerevisiae* strain (TMB4443) carrying the oxido-reductive xylose pathway from *S. stipitis* (Sandström et al. 2015). By expressing an acetoacetyl-CoA reductase dependent on the cofactor nicotinamide adenine dinucleotide (NADH) from *Allochromatium vinosum* in combination with an improved xylose pathway, it was shown possible to obtain anaerobic PHB production from xylose resulting in the highest PHB content per cell (14 wt %) reported so far for *S. cerevisiae* in the strain TMB4425 (de Las Heras et al. 2016).

Redirection of carbon flux into a desired product by nutrient limitation is a well-established strategy in several industrial processes, e.g. the citric acid fermentation (Anastassiadis et al. 2002; Yalcin et al. 2010). Growth limitation, caused by e.g. limiting the availability of nitrogen, in the presence of a carbon source, is a key factor for obtaining high PHB titers in natural producers (Wang and Lee 1997). In non-engineered *S. cerevisiae*, nitrogen limitation causes accumulation of other storage carbohydrates, primarily glycogen, but to some extent also trehalose (Lillie and Pringle 1980). The accumulation of glycogen and trehalose is proportional to the duration of the G_1_ phase of the growth cycle—interphase step where growth organelles and cell material are dividing but DNA replication is not taking place (Paalman et al. 2003). Within a given concentration range, glycogen confers a competitive advantage in growth over cells that do not accumulate this storage compound (Anderson and Tatchell 2001). As for trehalose, it is associated with stress protection, acting as a stabilizer of the different cell compartments under heat stress, for example Crowe and Chapman 1984; Hottiger et al. 1994). The accumulation of both carbohydrates is tightly regulated in yeast and confers survival advantages to the cell (François and Parrou 2001).

**Fig. 1:**
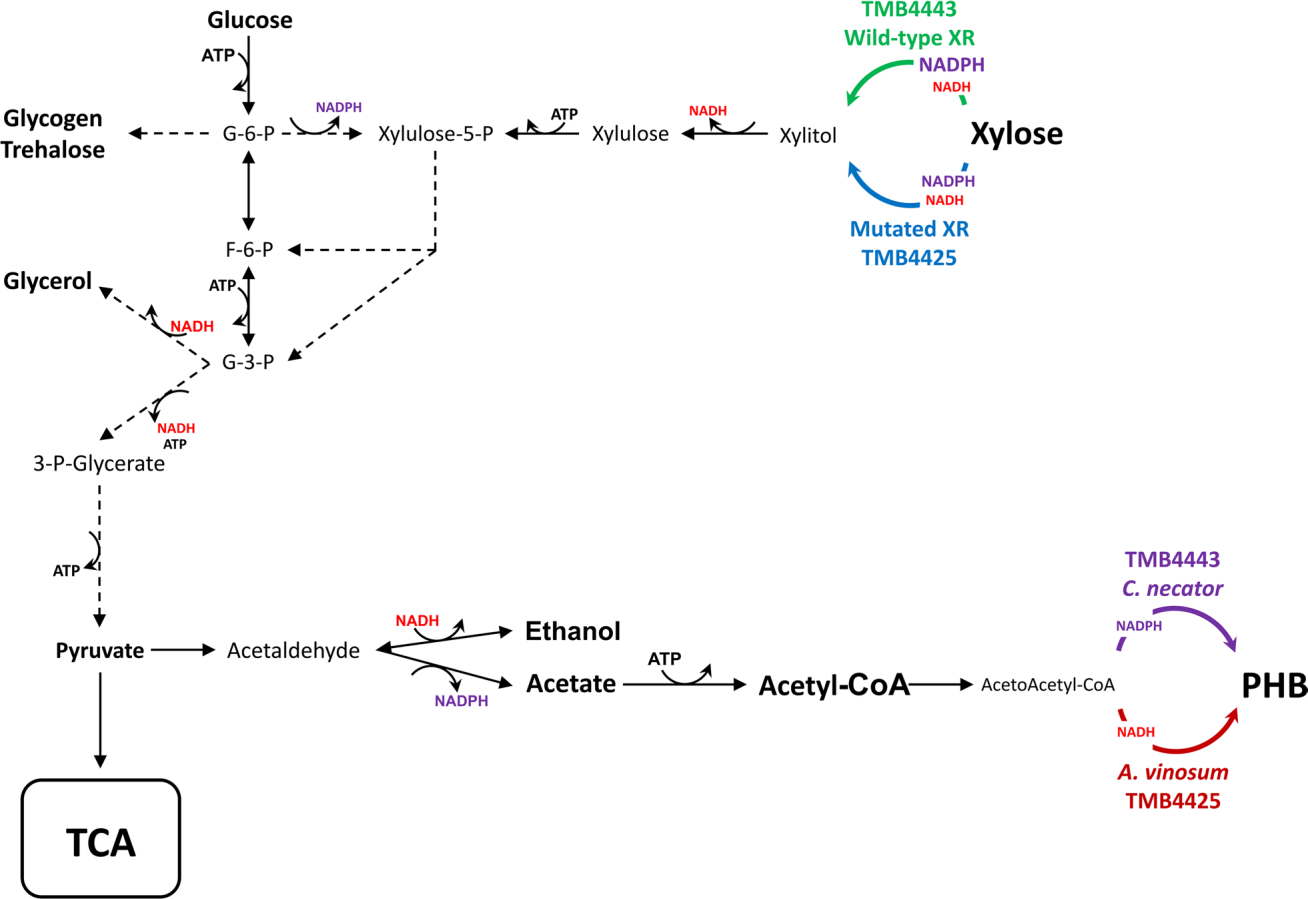
Metabolic map representing the main mechanisms that connect the xylose consumption pathway with the PHB producing pathway. Two alternative xylose reductases (XR) are represented: wild-type XR from *S. stipitis* present in TMB4443 and a mutated version of it that has different cofactor affinity present in TMB4425. The size of the size of the letters in the XR alternatives represent the preference for NADPH or NADH (bigger size → higher affinity). Two alternative acetoacetyl-CoA reductases that are part of the PHB producing pathway from acetyl-CoA are also shown: the first from *Cupriviadus necator* (present in TMB4443) and the second from *Allochromatium vinosum* (present in TMB4425). The dashed arrows represent more than one metabolic reaction in order to simplify the scheme

The recently described PHB production in *S. cerevisiae* from xylose together with the extensive on-going research on boosting the intracellular pool of the precursor acetyl-CoA and fine-tuning the redox state of the cell, opens up several routes for the optimization of the PHB formation. The aim of the present study was to specifically investigate the effect of nitrogen availability on the PHB accumulation of two strains of *S. cerevisiae* engineered for PHB production. Glucose or xylose were used as carbon sources, since these are likely to be present in various residue streams derived from lignocellulosic biomass. The two strains used, TMB4443 and TMB4425, were both able to consume xylose by an introduced two-step conversion of xylose to xylulose by xylose reductase (XR) and xylitol dehydrogenase (XDH) (de Las Heras et al. 2016; Sandström et al. 2015). However, the strains were based on expression of different heterologous xylose and acetoacetyl-CoA reductases, with different affinities for the co-factors NADH and NADPH (Fig. 1). Cultivation experiments were performed in media with different ratios between the carbon and the nitrogen source, and consumption of sugars as well as the accumulation of PHB, glycogen and trehalose were measured. To the best of our knowledge, the effect of nitrogen deficiency on PHB accumulation and its relation with the other storage carbon compounds found in *S. cerevisiae* has not been investigated yet.

## Materials and methods

### Strains, media and culture conditions

Genetically engineered strains of *S. cerevisiae*, namely TMB4443 (Sandström et al. 2015) and TMB4425 (de Las Heras et al. 2016) with the ability to accumulate PHB were used in this study. The PHB producing pathway in TMB4443 consists of the genes *PhaA*, *PhaB1* and *Phac1* from *C. necator* and harbors the wild-type XR-XDH pathway from *Scheffersomyces stipitis* (Sandström et al. 2015E). In TMB4425, the acetoacetyl-CoA reductase that is coded by *PhaB1* was replaced by an alternative NADH-dependent reductase from *Allochromatium vinosum* (de Las Heras et al. 2016). Another particular trait of TMB4425 is that harbors a mutated version of the XR instead of the wild-type, which confers a balance affinity for NADH and NADPH. TMB4444 was used as negative control since it lacks the PHB producing pathway (Sandström et al. 2015E). In all cases, the xylose consuming pathways were optimized by overexpressing the pentose phosphate pathway (de Las Heras et al. 2016; Sandström et al. 2015).

The pre-cultures were recovered by transferring a 10 µL loop of cells from the glycerol stocks stored at −80 °C into 5.0 mL of a growth medium containing 13.4 g yeast nitrogen base (YNB)/L, 50 mM potassium hydrogen phthalate buffer at pH 5.5 and was supplemented either with 50 g d-glucose/L or 50 g d-xylose/L, depending on the carbon source used in the further cultivations. Cells were grown in a 50 mL sterile conical centrifuge tube (Sarstedt AG & Co., Nümbrecht, Germany) and incubated at 30 °C and 180 rpm until the end of the exponential growth phase was reached.

Two different *Yeast Base* (YB) media were tested in the further aerobic cultivations and anaerobic fermentations: (1) *N*-*high*, which contained 13.4 g YNB without amino acids and ammonium sulfate/L, 10.0 g ammonium sulfate/L, and 50 mM potassium hydrogen phthalate buffer at pH 5.5 and (2) *N*-*def*, which contained 13.4 g YNB without amino acids and ammonium sulfate/L, 50 mM potassium hydrogen phthalate buffer at pH 5.5. Depending on the experimental design the growth media *N*-*high* and *N*-*def* were supplemented with either 50 g glucose/L (termed as *Glc*-*N*-*high* and *Glc*-*N*-*def*, respectively) or 50 g xylose/L (termed as *Xyl*-*N*-*high* and *Xyl*-*N*-*def*, respectively). In the case of the anaerobic fermentations, 0.01 g ergosterol/L and 0.42 g Tween 80/L were added to the medium.

### Cultivations and fermentations

#### PHB accumulation tests under high nitrogen availability

Yeast cell pellets obtained from the pre-cultures were centrifuged (4000 rpm, 5 min, 4 °C), resuspended and washed with sterile saline solution (0.9% NaCl). The cells were re-suspended in the cultivation medium and inoculated such that an initial optical density of 0.05 was obtained. The optical density was measured at a wavelength of 620 nm (OD_620nm_) by using an Ultrospec 2100 Pro spectrophotometer (Amersham Biosciences Corp., New Jersey, USA). Yeast strains were cultivated in 250 mL shake-flasks with baffles containing 50 mL medium for the aerobic cultivations. A sterile foam plug was used in order to keep the aerobicity during the experiment. In the case of anaerobic fermentations, 250 mL shake-flasks without baffles but with a rubber stopper and a glycerol trap were used. Nitrogen gas (N_2_) was continuously sparged with the aid of a sterile needle that was in contact with the liquid medium over the fermentation. The flasks were incubated in a shaking incubator operated at 30 °C and 180 rpm. TMB4443 was tested in *Glc*-*N*-*high* and *Xyl*-*N*-*high* under aerobic conditions, and only in *Glc*-*N*-*high* under anaerobic conditions. TMB4425 was evaluated in *Glc*-*N*-*high* and *Xyl*-*N*-*high* both under aerobic and anaerobic conditions. OD_620nm_ was followed overtime (Ultrospec 2100 Pro spectrophotometer, Amersham Biosciences, Corp., USA) and samples for cell dry weight (CDW), extracellular metabolites and PHB analysis were collected in technical duplicate. For each condition and strain evaluated, two biological replicates were performed.

#### PHB accumulation tests under nitrogen deficiency

Yeast cell pellets obtained from the pre-cultures were centrifuged (4000 rpm, 5 min, 4 °C), re-suspended and washed with 0.9% NaCl. An intermediate step for cell proliferation was made by growing the cells in 50 mL of either *Glc*-*N*-*high* or *Xyl*-*N*-*high* by inoculating with the washed cells such that initial OD_620 nm_ corresponding to 0.05 was achieved. The cultures were incubated at 30 °C and were subjected to shaking at 180 rpm. The cells were harvested by centrifugation at the end of the exponential growth phase and were washed and re-suspended subsequently. These re-suspended cells were inoculated at an initial OD_620 nm_ of 12.5, corresponding to approximately 4.0 g biomass/L. TMB4443 was cultivated aerobically in 250 mL shake-flasks with baffles containing 50 mL medium (*Glc*-*N*-*def*). A sterile foam plug was used to in order to keep the aerobicity during the experiment. In the case of anaerobic fermentations, TMB4425 was evaluated in 250 mL shake-flasks without baffles containing 50 mL of *Xyl*-*N*-*def* medium. The shake-flasks were equipped with a rubber stopper and a glycerol trap and N_2_ was continuously sparged with the aid of a sterile needle that was in contact with the liquid medium over the fermentation. For each condition and strain evaluated, two biological replicates were performed.

### Analytical assays

#### Biomass

Biomass was quantified by measuring CDW. CDW corresponding to the end-point of the cultivations was measured in duplicate by filtering a measured volume of the culture through a pre-weighed 0.45 µm pore size nitrocellulose filter (PALL, Michigan, USA). After the drying process the filters were equilibrated to room temperature in a desiccator and then weighed. Biomass was correlated to OD_620 nm_ by a single-point calibration, based on the final time point measurement.

#### Extracellular metabolites and PHB

Concentrations of glucose, xylose, xylitol, glycerol, acetate and ethanol were determined by high-performance liquid chromatography (HPLC, Waters, Massachusetts, USA). The compounds were separated using an Aminex HPX-87H ion exchange column (Bio-Rad, California, USA) preceded by a Micro-Guard Cation-H guard column (Bio-Rad, California, USA). Separation was performed at 45 °C with 5 mM H_2_SO_4_ as mobile phase at a flow rate of 0.6 mL/min. All compounds were quantified by refractive index detection (Waters, Massachusetts, USA). A seven-point calibration was made for each compound to determine the concentrations and each sample was analyzed at least in technical duplicate.

The quantification of PHB was based on the method described by Law and Slepecky, where the conversion of the polymer into crotonic acid is catalyzed by hot concentrated sulfuric acid (Law and Slepecky 1961). The extraction and analytical methods were followed as described Sandström et al. (2015).

#### Glycogen and trehalose

Glycogen and trehalose extraction was done simultaneously for each collected sample by using the method described by Parrou and François (1997) that is based on the alkaline digestion of yeast cells in 0.25 M Na_2_CO_3_ at 95 °C (Becker 1978).

Glycogen concentrations were determined using the Glycogen Assay Kit (#MAK016, Sigma-Aldrich, Missouri, USA). The samples analyzed corresponded to the initial (0 h) and end-points (170 h) of the experiments performed under nitrogen deficiency with the strains TMB4425 (*Xyl*-*N*-*def*), TMB4443 (*Glc*-*N*-*def* and *Xyl*-*N*-*def*) and TMB4444 (*Xyl*-*N*-*def*). The end-point samples corresponding to the assays performed in *Xyl*-*N*-*high* with TMB4425 and *Glc*-*N*-*high* with TMB4443 were also analyzed. Samples were diluted 50–100 times and analyzed in technical duplicates. The glycogen concentration was calculated from a six-point calibration curve.

Trehalose was determined by ultra-performance liquid chromatography (UPLC) (Waters, Massachusetts, USA). Separation was performed with the aid of a Waters UPLC BEH Amide 2.1 × 100 mm (Waters, Massachusetts, USA) column at 50 °C and 0.4 mL min^−1^. A gradient of concentrations of acetonitrile and aqueous solution of ammonium acetate was created according to the method recently described by Almqvist et al. (2016). Trehalose was quantified by evaporative light scattering detection (Waters, Massachusetts, USA).

## Results

### Effect of nitrogen availability on PHB production using glucose as sole C-source

The performance of *S. cerevisiae* TMB4443, having NADPH preference for xylose and PHB pathways, was evaluated under aerobic conditions to compare the effect of the absence or excess of nitrogen. As initial work indicated that TMB4443 accumulated higher intracellular concentrations of PHB when glucose was used instead of xylose (data not shown), glucose was chosen as the principle carbon source for TMB4443 in the assessment of effects of nitrogen availability.

**Fig. 2:**
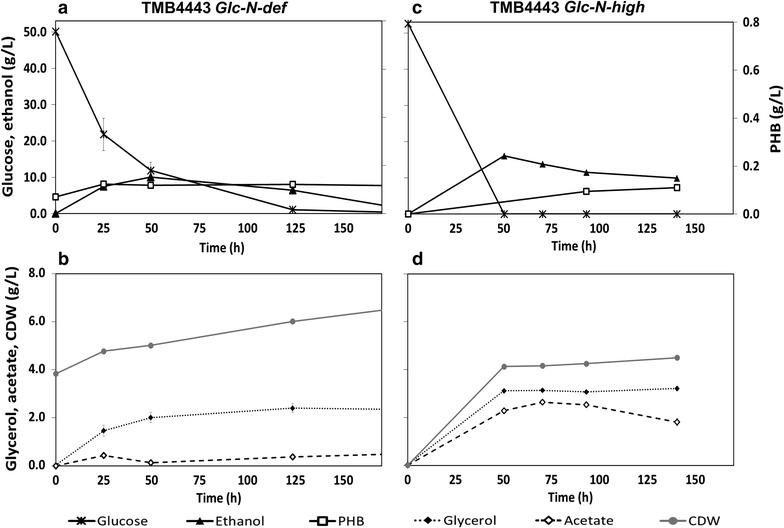
Metabolite profiles of *S. cerevisiae* TMB4443 during 170 h of cultivation under aerobic conditions in glucose defined medium. Glucose and ethanol profiles are shown under the absence (**a**) or the excess of nitrogen (**c**). **b**, **d** correspond to the *N*-*def* and *N*-*high* experiments, respectively, and show the variation of glycerol, acetate and CDW levels. Each set of graphs (**a** and **b** for *N*-*def*; **c** and **d** for *N*-*high*) corresponds to one biological replicate representative of the two that were performed

TMB4443 was able to consume all of the added glucose even in the absence of ammonium sulfate (*Glc*-*N*-*def*, Fig. 2a). However, upon addition of ammonium sulfate (*Glc*-*N*-*high,* Fig. 2c), the cells consumed glucose at a rate that was at least twofold higher, as glucose was depleted after 50 h of cultivation compared to approximately 125 h required by the cells in cultures lacking ammonium sulfate (*Glc*-*N*-*def,* Fig. 2a). In the case of *Glc*-*N*-*high*, where a low starting biomass of 0.01 g CDW/L was used, 4.8 ± 0.4 g CDW/L was formed (Fig. 2d), indicating that the cell growth was not limited by the nitrogen source or other nutrients. The same intended initial biomass was tried in the *Glc*-*N*-*def* experiments but no significant growth was observed (data not shown). For this reason, high initial cell density (3.8 g CDW/L) was used for the *N*-*def* experiments. Initially, glucose was rapidly converted mainly into ethanol which reached its maximum concentration after 50 h of cultivation. Ethanol production under aerobic conditions with high residual glucose concentration was expected since *S. cerevisiae* is a Crabtree-positive yeast (De Deken 1966). At this time point, where the titer of PHB was 0.12 g PHB/L, PHB accumulation stopped. Subsequent ethanol consumption did not lead to further PHB accumulation (Fig. 2a). In *Glc*-*N*-*high* conditions, the analysed metabolites reached higher titers. The maximum glycerol and ethanol titers were observed after 50 h of cultivation. The maximum acetate titer, achieved within 70 h, was about sixfold higher than that achieved with *Glc*-*N*-*def* assay. The final PHB titer was 0.11 g PHB/L and since the initial biomass concentration was low, no PHB was detected at the beginning of the cultivations (Fig. 2c).

**Fig. 3:**
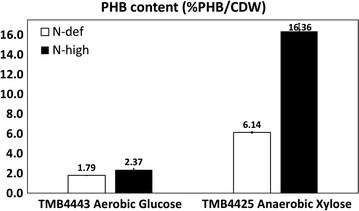
Effect of absence or excess of a nitrogen source on the PHB content by the strains TMB4443 and TMB4425. TMB4443 was evaluated under aerobic conditions and using glucose as sole carbon source. TMB4425 was tested in anaerobic conditions and with xylose as sole carbon source. The values shown for each condition correspond to an average of the two independent biological replicates performed

Although similar final PHB titers were obtained at the end of both experiments, a substantial fraction of the PHB was present in the biomass already at the start in the case of nitrogen limitation because the cells were pre-grown in a medium containing glucose with excess of ammonium sulfate – condition that favors PHB accumulation (Fig. 2c). The net PHB production (PHB_final concentration_ − PHB_initial concentration_) was only 0.05 g PHB/L during the consumption of the glucose under nitrogen deficient conditions and it should be compared to 0.11 g PHB/L obtained in the presence of ammonium sulfate (Fig. 3).This was accompanied by a higher PHB content (% PHB/CDW) when ammonium sulfate was supplied (Fig. 3; Table 1).

**Table 1:** Physiological characterization related parameters of the PHB-producing strains TMB4443 and TMB4425 under aerobic and anaerobic conditions, respectively

Strain	C-source	Nitrogen supply	Aeration	Initial CDW (g/L)	Yields						PHB titer (g/L)	PHB content (% PHB/CDW)
					Y_sX_ (g/g C-source)	Y_sXylOH_ (g/g C-source)	Y_sGlyOH_ (g/g C-source)	Y_sAcet_ (g/g C-source)	Y_sEtOH_ (g/g C-source)	Y_sPHB_ (mg/g C-source)		
TMB4443	Glucose	No	Aerobic	3.8 ± 0.0	0.06 ± 0.00	0.00 ± 0.00	0.04 ± 0.01	0.01 ± 0.00	0.04 ± 0.01	1.0 ± 0.1	0.12 ± 0.00	1.8 ± 0.0
TMB4443	Glucose	Yes	Aerobic	0.01 ± 0.00	0.09 ± 0.01	0.00 ± 0.00	0.05 ± 0.01	0.03 ± 0.01	0.14 ± 0.07	2.12 ± 0.04	0.11 ± 0.00	2.4 ± 0.0
TMB4425	Xylose	No	Anaerobic	4.1 ± 0.2	0.06 ± 0.01	0.02 ± 0.00	0.09 ± 0.01	0.01 ± 0.00	0.01 ± 0.01	8.3 ± 0.8	0.43 ± 0.00	6.1 ± 0.1
TMB4425	Xylose	Yes	Anaerobic	0.02 ± 0.00	0.09 ± 0.00	0.04 ± 0.00	0.04 ± 0.00	0.01 ± 0.00	0.21 ± 0.03	13.8 ± 0.6	0.73 ± 0.00	16.4 ± 0.8

Cell dry weight (CDW), volumetric yields and titers were calculated from a single time-point corresponding to the end of the experiment (varied from 141 to 172 h). Values reported in the table represent the mean ± SD of at least two independent biological replicates. Glucose or xylose was used as sole C-source

Y_SX_: biomass yield on C-source, Y_SXylOH_: xylitol yield on C-source, Y_SGlyOH_: glycerol yield on C-source, Y_SAcet_: acetate yield on C-source, Y_SEtOH_: ethanol yield on C-source and Y_SPHB_: PHB yield on C-source. PHB titer: final PHB concentration. PHB content: percentage of PHB accumulated in the biomass formed

### Effect of nitrogen availability on PHB production using xylose as sole C-source

**Fig. 4:**
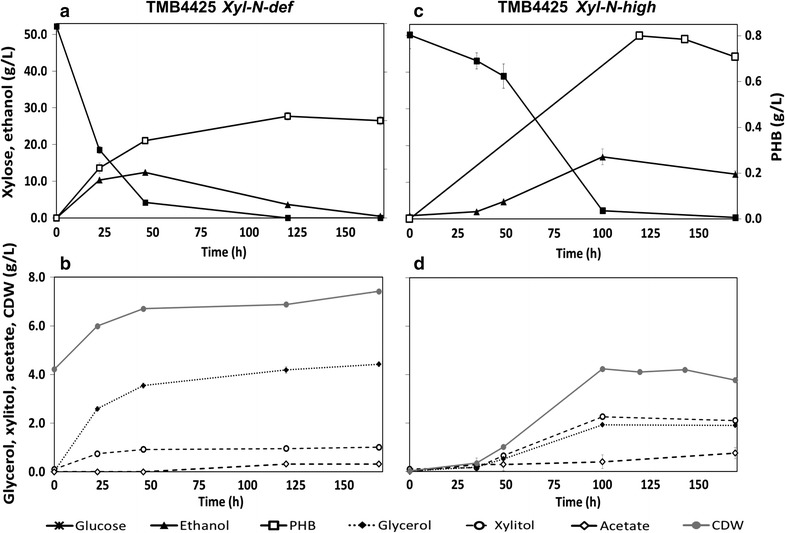
Metabolite profiles of *S. cerevisiae* TMB4425 during 170 h of cultivation under anaerobic conditions in xylose defined medium. Xylose and ethanol profiles are shown under the absence (Fig. 2a) or the excess of nitrogen (Fig. 2c). Figures 2b, d correspond to the *N*-*def* and *N*-*high* experiments, respectively, and show the variation of glycerol, xylitol, acetate and cell dry weight (CDW) levels. Each set of graphs (**a** and **b** for *N*-*def*; **c** and **d** for *N*-*high*) corresponds to one biological replicate representative of the two that were performed

The effect of nitrogen availability on PHB accumulation was also evaluated in *S. cerevisiae* TMB4425, which has a preference for NADH as a reducing co-factor in both the added xylose and PHB pathways. Similar to TMB4443, TMB4425 was also characterized in a medium containing either glucose or xylose and under aerobic or anaerobic conditions. As TMB4425 accumulated more PHB when grown on xylose under anaerobic conditions (data not shown), only anaerobic conditions were applied while evaluating the effect of nitrogen availability on the PHB accumulation by TMB4425 (Fig. 4).

As established earlier a high starting cell density was needed in the *N*-*def* experiments which allowed xylose to be depleted in *Xyl*-*N*-*def* (Fig. 4a). The cells accumulated PHB during growth on xylose, reaching a final and maximum PHB titer of 0.43 ± 0.04 g PHB/L. In contrast to the *Glc*-*N*-*def* experiment, no PHB was detected at the beginning of *Xyl*-*N*-*def*. This was due to the fact that cells were pre-grown in conditions unfavorable to PHB accumulation i.e. on xylose in aerobic conditions. The initial xylose consumption resulted mostly in biomass and ethanol formation, but also in low amounts of glycerol and xylitol. At 50 h, ethanol titer peaked after which it started declining. Ethanol consumption under anaerobic conditions is not feasible in *S. cerevisiae* due to a redox imbalance and low availability of ATP (Henningsen et al. 2015), but the decrease was instead due to evaporation resulting from the continuous sparging with nitrogen gas. Biomass formation and glycerol production continued until the end of the experiment (Fig. 4b).

When ammonium sulfate was added, *Xyl*-*N*-*high*, a low initial biomass inoculum was used which gave a lower volumetric sugar uptake rate than in the previous case (Fig. 4c). However, xylose uptake rate increased and xylose was nearly depleted after 100 h of fermentation.

Xylose consumption resulted mainly in biomass and ethanol formation (Fig. 4). The presence of a nitrogen source resulted in higher xylitol than glycerol accumulation (Fig. 4d), in contrast to what was observed in *Xyl*-*N*-*def* (Fig. 4b). The maximum PHB titer was achieved after 120 h (0.81 ± 0.01 g PHB/L), and then it slightly decreased until the end of the fermentation. Since PHB accumulates intracellularly, any cell lysis might release PHB in the medium, which will not be accounted for in the PHB extraction assay that relies on the analysis of the cell pellet. Thus, it is possible that cell lysis caused an underestimation of the PHB levels at the end-point.

The addition of ammonium sulfate thus resulted in a more efficient PHB production, giving a higher final PHB concentration. Importantly, the intracellular PHB concentration (%PHB/CDW) was 2.7-fold higher in the medium containing nitrogen than in the nitrogen deficient medium (Fig. 3; Table 1).

### Intracellular concentrations of glycogen, trehalose and PHB

Glycogen, PHB and trehalose were determined for TMB4425 and TMB4443. As a control, strain TMB4444 that lacks the PHB producing pathway was also evaluated.

The initial samples (0 h) were taken from the experiments performed with nitrogen deficient media (*N*-*def*), since accurate quantification of glycogen, trehalose and PHB required a sufficient amount of biomass (approximately 10^6^ cells). Therefore the initial samples reflect the intracellular accumulation of the pre-culture, i.e. a medium with high nitrogen content.

**Fig. 5:**
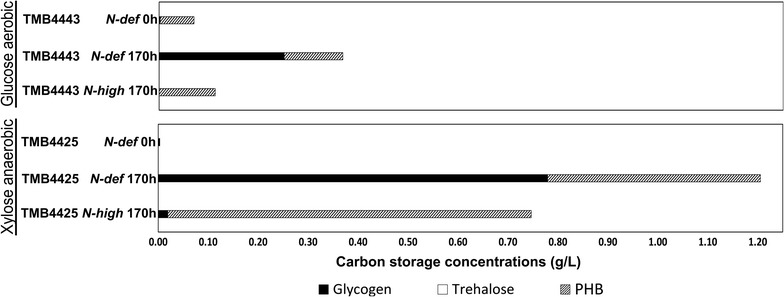
Cumulative accumulation of the carbon storage compounds (glycogen, trehalose and PHB) at the beginning (0 h) and end of the cultivations (170 h) for strains TMB4443 (aerobic, glucose) and TMB4425 (anaerobic, xylose)

In the control strain TMB4444, only glycogen was detected as carbon storage compound (data not shown), whereas PHB and glycogen were found in TMB4425 and TMB4443. In those strains, the distribution of the intracellular concentrations of the three storage molecules was compared (Fig. 5). The inoculum pre-culture used for the *N*-*def* experiments had a very low glycogen content and 97–98% of the carbon storage was PHB. During consumption of sugars in the absence of a nitrogen source, there was an increase in the glycogen accumulation, reaching 0.25 ± 0.00 and 0.78 ± 0.07 g glycogen/L in glucose and xylose, respectively. After normalization, these glycogen levels correspond to 62 and 93% of the final total storage carbon, respectively. In contrast, only PHB was found as storage compound in the experiments in which both ammonium sulfate and glucose were supplied. For strain TMB4425, 98% of the storage polymers was PHB under anaerobic conditions with xylose as carbon source and in the presence of a nitrogen source after 170 h (Fig. 5). The pre-culture of TMB4425 did not show PHB accumulation (i.e. the value at 0 h), i.e. there was no polymer accumulated when cells were pre-grown aerobically prior to inoculation into their respective nitrogen deficient media. This result correlates well with metabolite profiles shown in Fig. 4a.

The absence of ammonium sulfate during 170 h induced glycogen production in TMB4443 (*Glc*-*N*-*def*) and TMB4425 (*Xyl*-*N*-*def*), suggesting that this effect was not strain or substrate dependent. In order to confirm this hypothesis, TMB4443 was also evaluated in xylose media under aerobic conditions to determine if a different carbon source would result in a different pattern. In this case, after 170 h more than 90% of the total carbon storage was glycogen (data not shown).

## Discussion

The intracellular PHB levels reported here are clearly lower than the intracellular levels obtained by some natural prokaryotic PHB producers (PHB content above 50%). However, those levels were not obtained using xylose as a carbon source. Furthermore, the robustness and inhibitor tolerance of *S. cerevisiae* is an advantage for utilization of xylose-rich waste streams, e.g. from the paper industry. In the present study, we show that the removal of nitrogen from the culture medium has a negative impact on the PHB content in *S. cerevisiae*. In previous studies where a decreased amount of nitrogen in the medium did not improve PHB accumulation in *S. cerevisiae* (Carlson and Srienc 2006) or in engineered *Komagataella pastoris* (formerly known as *Pichia pastoris*) (Vijayasankaran et al. 2005), it was speculated that the low nitrogen level would affect the level of recombinant protein production (Carlson and Srienc 2006). Here we observe that, under nitrogen starvation, PHB production also directly competes for carbon with glycogen formation and that this effect is independent of the engineering strategy, aeration and carbon source.

The availability of glucose-6-phosphate (G6P) that diverts directly from glucose, is considered to be the key for the regulation of glycogen and trehalose accumulation in *S. cerevisiae* (François and Parrou 2001). Here glycogen accumulation in the absence of nitrogen is observed not only on glucose but also on xylose, with both strains and under aerobic or anaerobic conditions. This suggests that at least one of the glycolytic intermediates that are formed from xylose conversion into central metabolism, namely fructose-6-phosphate (F6P) and/or glyceraldehyde-3-phosphate (G3P) (Fig. 1), are channeled upwards to G6P via gluconeogenic enzymes including the enzyme phosphoglucose isomerase (PGI) that interconverts G6P and F6P. This is in agreement with transcriptomics data indicating increased transcription of genes involved on gluconeogenesis on xylose (Jin et al. 2004; Runquist et al. 2009). A decrease in PGI activity has previously been shown to negatively impact xylose uptake (Jeppsson et al. 2002) and it was then speculated that the decreased NADPH, arising from low G6P being channeled to the oxidative branch of the pentose phosphate pathway (PPP) (Fig. 1), directly affected the NADPH-dependent xylose reduction to xylitol by xylose reductase (XR). Here we observe glycogen formation under nitrogen starvation and also for a strain carrying a XR with a balanced cofactor preference for NADH and NADPH, which implies that the gluconeogenic flux is probably not regulated by the NADPH need of the XR enzyme nor by the NADPH need for biomass synthesis. Instead it might constitute an overflow resulting from the deregulation of gluconeogenic enzymes. However, this carbon redirection will result in less ATP being generated per xylose. Reduced ATP and enhanced G6P will promote glycogen synthesis since G6P is a stimulator and ATP an inhibitor of the glycogen synthase (François et al. 1988).

In contrast to previous reports with recombinant PHB producing *S. cerevisiae* (Kocharin et al. 2012; Vijayasankaran et al. 2005), in the present study PHB production was observed during the growth phase on both glucose and xylose. In the evaluated strain, the PHB genes were expressed under constitutive promoters, that is without any regulation, as opposed to what happens in natural producers (Pötter et al. 2002). The presence of PHB enzymes under growing conditions together with the availability of precursors such as acetyl-CoA and NAD(P)H, which is favored by the high levels of nitrogen, explains the PHB accumulation during growth. Still, PHB competes with biomass synthesis for these precursors, so the level of PHB accumulation is restricted. In the bacteria that naturally accumulate PHB, this polymer can be re-utilized as a carbon source in starvation periods. However, as yeast does not contain enzymes necessary for degradation of PHB, PHB accumulates as a true “by-product” of the metabolism since the cells do not directly benefit from its accumulation.

Notwithstanding the differences in the fermentation conditions, the PHB pathway present in the strain TMB4425 was demonstrated to be more efficient for PHB production in yeast than the one present in TMB4443. This result is in agreement with the recent observations of Muñoz de las Heras and co-workers (2016), where it was shown that the replacement of the acetoacetyl-CoA reductase (*PhaB*) from *C. necator* by the NADH-dependent reductase from the anoxygenic purple sulfur bacteria *A. vinosum*, resulted in higher PHB content and titer under oxygen-limited conditions (de Las Heras et al. 2016). Anaerobic bioprocesses are usually associated with certain advantages for industrial applications due to the lower operating costs for aeration than those required for fully aerobic processes (Kozak et al. 2014; Weusthuis et al. 2011). Under anaerobic conditions, NADH is generated in the glycolysis of *S. cerevisiae* and as a result of anabolic reactions. Reoxidation to NAD^+^ is obtained through reduction of acetaldehyde to ethanol as well as by reduction of dihydroxyacetone phosphate to glycerol-3-phosphate (which is subsequently dephosphorylated to glycerol) in wild-type strains. In the strain holding the NADH-coupled acetoacetyl-CoA reductase, the PHB pathway can also serve to reoxidize NADH and maintain the co-factor balance in the cell. The decreased need for NADPH in the PHB pathway of TMB4425, may also be beneficial since it does not compete for NADPH needed for biosynthetic purposes.

In conclusion, the current study demonstrates that PHB accumulation in the evaluated *S. cerevisiae* strains with heterologous PHB formation pathways was higher in the presence of a nitrogen source in the medium. The attempt to redirect the carbon flux towards PHB by limiting cell growth clearly competes with the tightly regulated natural mechanism of carbon storage of *S. cerevisiae* under nitrogen limitation. Interestingly, glycogen accumulation from xylose was detected, indicating a significant gluconeogenic activity on this pentose. The NADH-coupled PHB producing pathway, in combination with a mutated XR in the strain TMB4425, was the most efficient pathway for PHB production from xylose, resulting in an intracellular PHB content as high as 16%.

## Abbreviations

Acetyl-CoA: acetyl coenzyme A
CDW: cell dry weight
F6P: fructose-6-phosphate
Glc: glucose
G3P: glyceraldehyde-3-phosphate
G6P: glucose-6-phosphate
HPLC: high-performance liquid chromatography
NAD^+^/H: nicotinamide adenine dinucleotide
NADP^+^/H: nicotinamide adenine dinucleotide phosphate
*N*-*high*: nitrogen excess medium
*N*-*def*: nitrogen deficient medium
PGI: phosphoglucose isomerase
PHA: polyhydroxyalkanoate
PHB: polyhydroxybutyrate
PPP: pentose phosphate pathway
SSL: spent sulfite liquor
wt: wild-type
XDH: xylitol dehydrogenase
XK: xylulokinase
XR: xylose reductase
Xyl: xylose
